# Characterization of Skeletal Muscle Regeneration Revealed a Novel Growth Network Induced by Molecular Acupuncture-like Transfection

**DOI:** 10.3390/biom14030363

**Published:** 2024-03-19

**Authors:** Ernő Zádor

**Affiliations:** Institute of Biochemistry, Albert Szent-Györgyi Faculty of Medicine, University of Szeged, Dóm tér 9, H-6720 Szeged, Hungary; zador.erno@med.u-szeged.hu

**Keywords:** skeletal muscle, regeneration, molecular acupuncture, SERCA, Ras, in vivo transfection

## Abstract

The low efficiency of in vivo transfection of a few fibres revealed a novel tissue network that temporally amplified growth stimulation in the entire regenerating rat soleus muscle. This acupuncture-like effect was demonstrated when the fibres began to grow after complete fibre degradation, synchronous inflammation, myoblast and myotube formation. Neonatal sarcoplasmic/endoplasmic reticulum ATPase (SERCA1b) was first detected in this system. The neonatal, fast and slow SERCA isoforms displayed consequent changes with innervation and differentiation, recapitulating events in muscle development. In vivo transfection of myotubes with plasmids expressing dominant negative Ras or a calcineurin inhibitor peptide (Cain/cabin) proved that expression of the slow myosin heavy chain and the slow muscle type SERCA2a are differentially regulated. In vivo transfection of a few nuclei of myotubes with dnRas or SERCA1b shRNA stimulated fibre size growth in the whole regenerating muscle but only until the full size had been reached. Growth stimulation by Ras and SERCA1b antisense was abolished by co-transfection of Cain or with perimuscular injection of IL4 antibody. This revealed a novel signalling network resembling scale-free networks which, starting from transfected fibre myonuclei as “hubs”, can amplify growth stimulation uniformly in the entire regenerating muscle.

## 1. Introduction

The skeletal muscle is a favourite subject of differentiation studies, not just because it is the largest tissue in mammals, contributing around 40% of body weight [1], but also due to its importance in movement and healthy life [2]. To maintain the integrity of such an organ, it is crucial that the skeletal muscle have remarkable plasticity and be trained to respond to environmental constraints and pathological conditions [3,4]. The most dramatic response is needed during regeneration when the muscle rebuilds itself after damage [5]. The main sources of regeneration are the muscle stem cells singled out from the satellite cells located between the basal lamina and the sarcolemma [6,7,8]. However, satellite cells behave differently during regeneration than during hypertrophy [9]. During extensive muscle damage, bone marrow cells [10,11,12] and non-muscle-resident fibroblasts [13,14] may also transform into myoblasts and contribute to myotubes at the beginning of and later during the process. Other stem cells called fibro-adipogenic progenitors can regulate the activity of muscle satellite cells [15,16]. A number of experimental models have been developed for muscle regeneration, but only a few are based on completely damaged muscle fibres [17]. Regeneration is more even after complete fibre degradation [18], although the kinetics of change does not seem to depend on the extent of damage [19]. If fibre degradation is partial, inflammation is sustained by type I macrophages that suppress myoblast formation [20,21]. Experimental regeneration can be induced using local anaesthetics or naturally occurring toxins like snake venoms [22,23]. Bupivacaine is probably one of the most often used local anaesthetics for this purpose [24,25], while notexin from the Australian tiger snake [26] is a frequently applied venom. However, notexin induces more complete muscle damage in mouse tibialis anterior (TA) compared with bupivacaine [27]. It was also found that rat soleus regenerates with a 1–2 day delay after intramuscular injection of notexin compared with bupivacaine [28,29]. This is because notexin results in more extensive muscle fibre damage compared with bupivacaine [27].

In the past few years, significant progress has been made in understanding the mechanism of the notexin effect [30]. This A2 phospholipase (PLA2) analogue toxin binds to unidentified receptors on muscle fibres and neuromuscular junctions, causes sarcolemma damage and starts perturbations that elevate sarcoplasmic Ca^2+^ levels [31,32]. The myotoxic effect is fairly selective for the type I and type IIA fibres and apparently spares early differentiated cells like satellite cells, myoblast precursors and myoblasts [18]. The different pathological consequences are probably not due to different mechanisms of cell toxicity but rather to the intrinsic physiological and anatomical properties of the targeted cells [31]. Accordingly, crotoxin, a myotoxin similar to notexin, can be antagonised by cyclosporine A (CSA). This can be interrupted so that CSA inhibits the opening of the transiently permeable pores of the inner membrane of the mitochondria [31]. Because oxidative type I and type IIA muscle fibres are more dependent on mitochondria than type IIb and type IIx/d glycolytic fibres, they are more vulnerable to PLA2 toxins. Notexin can induce muscle necrosis relatively far away from the site of administration but it does not appear to cross the blood–brain barrier, explaining why it can induce muscle regeneration so effectively after subcutaneous injection [33]. Notexin, in contrast to viper venoms [34], does not induce extensive haemorrhage since it leaves the blood vessels, the extracellular tissue and the nerves relatively intact; each of these promotes even regeneration [35].

Our aim was to establish an experimental system of complete regeneration in order to monitor developmental events. We intended to align the obtained information with the pattern of in vivo muscle differentiation. Myotube formation and innervation were considered crucial landmarks. The synchrony of events was studied using morphological, biochemical and molecular methods. Whole extracts of muscles from each stage of regeneration were studied with practically no contamination of non-regenerating or out-of-stage regenerating tissues. Complete necrosis and the subsequent regeneration were achieved via intramuscular injection of a ten times higher dose of purified snake venom (Sigma) than was administered subcutaneously in earlier reports [33] reviewed in [36]. The treatment induced full regeneration of the soleus muscle of three-month-old male Wistar rats weighing 300–360 g.

The purpose of this review was to highlight the timely importance of the regeneration system revisited here. The study was divided into descriptive and manipulating parts. The descriptive part established an in vivo system to measure fibre growth reliably to screen the effects of manipulations such as antisense treatment and in vivo transfection. It is worth emphasizing that no reports on trying to achieve synchrony of consequent events during the entire muscle regeneration process were found in the literature. As some of the studies are twenty-something years old, newer citations from the latest period were collected from PubMed and Google Search to correspond with the updates. These searches also revealed a similar growth network; therefore, it may be worthwhile to revisit this regeneration system in rat soleus from a newer, bioinformatics perspective.

## 2. Muscle Degradation, Inflammation and Increase in TNF-α Levels

**Figure 1:**
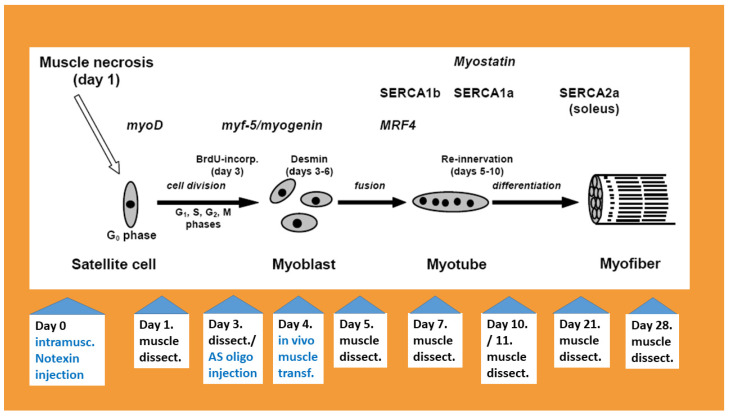
Schematic summary of the studied gene expression during rat soleus regeneration. Briefly, inflammation and fibre degradation occur after intramuscular injection of notexin. The satellite cells are activated and transform into muscle precursor cells and then into myoblasts. The myoblasts fuse into myotubes, become innervated and differentiate into muscle fibres. MRF, SERCA, myostatin and TNF-α indicate maximum levels of mRNAs/proteins on the time scale of characteristic morphological changes during regeneration. Note the consecutive neonatal (SERCA1b), fast (SERCA1a) and slow type (SERCA2a) calcium pump expressions and that SERCA2a remains the dominant form in the slow type soleus until the end of regeneration. The lower part of the figure shows a flowchart of the treatments and sample collection (muscle dissection) for RT-PCR, immunoblots, haematoxylin–eosin staining and immunohistochemistry. Antisense oligo injection was performed after the 3rd day and transfections to myotubes were performed after the 4th day of notexin injection.

After 6–24 h of intramuscular injection of an appropriate amount of snake venom, the solei of 300–360 g male Wistar rats became swollen and lost their red colour. This was probably due to the inflammation and degradation of myoglobin in mitochondria-rich fibres, which were the most sensitive to Ca^2+^ entry through the sarcolemma [31,32,37]. The venom toxins bind to a receptor on the sarcolemma and tissue damage is achieved through amplification of the initial effect via signal networks [38]. As the rat soleus muscle contains about 70% type I, 28% type IIA and only 1–2% type IIx fibres [39], this meant near total fibre degradation. Muscle pallor and weight loss lasted until day 4–5 of regeneration; the colour was restored by day 5 but the weight was regained only around day 28 of regeneration [40]. Microscopic changes were most evident on transversal sections of the necrotised-regenerating muscle; the fibre outline faded after 6 h and practically diminished 3 days after venom administration. A number of mononucleated cells populated endomysial sites and occurred throughout the cross-sections of the fibres [18]. In the beginning, when the optimal dose of snake venom was established, transversal sections were taken along the spindle-shaped soleus and scanned from proximal to distal tendons in order to check the completeness of muscle degradation. This was also confirmed by the dramatic decline or lack of detection of muscle-fibre-specific transcripts and proteins (i.e., MyHC-s, SERCA-s and skeletal actin) in the whole muscle homogenate [28,40,41]. However, blood vessels and the endomysia outline were partially preserved until 3 days after toxin administration [28]. A regeneration scheme showing the markers and flowchart of samples is presented in Figure 1.

In contrast to the decline in muscle-specific proteins, inflammation markers dramatically increased with muscle necrosis. The lymphocyte/macrophage-specific protein MAC 387 increased after 6 h, reaching a maximum 3 days after toxin injection based on immunoblot of whole muscle extracts [42]. This marker then gradually declined until 28 days after venom injection but remained above the normal level [42]. The persistence of MAC 387 indicated that lymphocytes/macrophages apparently accompanied muscle regeneration long after necrosis and probably influenced the autocrine–paracrine environment when regeneration looked macro- and microscopically complete [43]. Tumour necrosis factor α (TNF-α), a pleiotropic pro-inflammatory cytokine, increased about two orders of magnitude at the mRNA and protein levels. Comparison of the position of desmin-positive muscle cells and the in situ hybridization of TNF-α mRNA on cross-sections showed that the cytokine was not expressed in myoblasts and myotubes on day four of regeneration when its level was still around the maximum. The lack of TNF-α expression in muscle myoblasts and myotubes was supported by the absence of its transcript in differentiating in vitro cultures of muscle cells like C2C12 and BC3H1; however, the mRNAs of TNF receptors were expressed in these cell lines. During in vivo muscle regeneration, the change in TNF-α was paralleled by an increase in TNFR-60 but not TNFR-80 mRNA levels, suggesting that the smaller receptor has a primary role in the TNF-α response. The role of this cytokine in muscle regeneration is complex. It has been shown that TNF-α acts on muscle cells, just like in other tissues, through more than one pathway [44,45]. At high TNF-α levels, one of these pathways inhibits myogenesis activating NF-χB, which translocates to the nucleus and induces iNOS, IL-1 and TNF-α [46]. Another pathway prevents differentiation through the destabilization of myoD [47]. This leads to pro-proliferative and differentiation-suppressing changes [48,49]. The other pathway involves p38 kinase, which phosphorylates essential myogenic transcription factors like MyoD, myogenin and MEF-2 and therefore promotes differentiation to myotubes [50,51]. The latter occurs at low TNF concentrations. TNF-α is also a chemoattractant of satellite cells and mesangioblasts, which contribute to regeneration in dystrophic muscle [51,52]. The balance between inflammation and regeneration might be essential for in vivo muscle regeneration [53]. In a preliminary study, Remicade (cV1q), a drug containing chimeric antibodies against TNF-α, was administered subcutaneously and perimuscularly to the solei of 300–360 g male Wistar rats immediately after venom injection [54]. Remicade treatment increased fibre size and the intensity of desmin-specific immunostaining at 4 days compared to the control regenerating muscle. This supported the conclusion that TNF-α inhibits early events of muscle regeneration, including the formation and growth of myotubes/primitive fibres. This was in agreement with the ameliorating effect of Remicade on skeletal muscle fibrosis in mdx mice, where, however, it also had a negative impact on cardiac function [55]. Myostatin, the first myokine to be discovered, also appeared in this system, based on its growth inhibitory role, suppressed satellite cell activation [56], and later increasingly maintained levels with differentiation [57].

## 3. Myogenic Regulatory Factors

Skeletal muscle regeneration characteristically includes the selection of cells with mesodermal origin and their transformation into myoblasts [58]. The discovery of the myogenic regulatory factors (MRFs) in the myoD family led to a breakthrough in our understanding of this process. The four main members of this family—myoD (myf-3 in humans), myf-5, myogenin (myf-4 in humans) and MRF4 (herculin or myf-6)—were found to transform fibroblasts or other non-muscle-like cells into myoblasts [59,60,61,62]. The MRFs belong to the group of “basic-helix-loop-helix” (bHLH) transcription factor proteins that bind the E-box (CANNTAG) with their basic domain and then form homodimers or heterodimers with each other or (in most cells) with non-myogenic E proteins [63,64,65]. Indeed, the E-box is present in the regulatory region of a number of muscle-specific genes like the alpha subunit of nicotinic acetylcholine receptor, the myosin light chain, alpha-actin, desmin, troponin I and creatine kinase and is needed for muscle-specific expression [66,67,68]. The different roles of MRFs can be assumed based on their spatial and temporal expressions and the diverse phenotypes of their knock-out mutants [61,69]. The specificity of MRFs can be determined based on the interactions of their transactivation domains with other transcription factors. MyoD and myf-5 play an important role in transforming muscle precursor cells of the somite into myoblasts during early embryogenesis of mammals by activating chromatin loci of muscle-specific genes [67,70,71,72].

Describing the mRNA levels of myogenic regulatory factors during muscle regeneration is of interest; as far as I know, our work was the first to do this [18]. Originally, it was aimed at finding homology between muscle regeneration and embryonic myogenesis and revealing differences between slow (soleus) and fast (EDL) muscle regeneration. In soleus regeneration, as expected, myoD increased on day 1, reached a maximum on day 3 and then gradually decreased to near-normal levels by day 21. Myf-5 and myogenin mRNA levels also decreased on day 1, increased to their maximum levels on day 3 and then decreased gradually. The decline in MRF4 mRNA was more dramatic compared with that of the other factors and to the normal muscle level on day 1 but was restored by day 5 of regeneration. This shows that MRF4 is more bound to myotubes/fibres than to myoblasts. Interestingly, compared with the soleus, EDL muscle regeneration did not show major differences in the changes in myoD, myf-5 and myogenin mRNA levels. However, MRF4 did not decrease as significantly as in the soleus, most likely because EDL regeneration was less synchronous and had few degraded/partially damaged large fibres (probably types IIb and IIx) [18]. Additionally, the timing of the expression of myogenic factors at the resolution studied in our work was largely similar to the order (myoD/myf-5, myogenin, MRF4) reported during myogenesis [68,73]. A remarkable difference was observed in the level of myf-5 mRNA, which increased during myogenesis at about the same time as the myoD messenger [63] but declined in our regeneration system, in contrast to that of myoD. It has been reported that myoD knock-out mice show slightly delayed but otherwise quite normal muscle development. This could happen because increased myf-5 expression, which has the same cell specificity, complemented myoD function [69]. Such altered regulation of myf-5 and myoD might also occur during muscle regeneration. The other explanation for the decreased myf-5 messenger level might be that the satellite cells expressing myf-5 [74,75] were transformed into muscle progenitor cells and myoblasts that then predominantly expressed myoD.

The myoD protein followed its messenger in the regenerating soleus and showed a 6x increase compared to the normal level, reaching a maximum on the 3rd day after venom injection. This was paralleled by the maximum level of BrdU incorporated into the nucleus, implying that the proliferating cells, probably myoblasts, expressed the transcription factor. MyoD expression was disrupted by injecting antisense oligonucleotides into the muscle on the 3rd day of regeneration [76]. The antisense dramatically decreased mRNA and protein levels of myoD one hour after treatment; the myoD protein was about 3-fold less in the isolated myonuclei compared to the control regenerating soleus injected with scrambled oligonucleotide. The progress of regeneration was also delayed by this treatment, as judged two days later based on desmin expression, myotube formation and the number of primitive endplates [76,77]. This was somewhat surprising since myoD was reported to have a relatively quick turnover in cell culture [78,79]. The antisense oligo started to degrade after four hours and the myoD mRNA level was also restored by that time [76]. However, the antisense still worked, probably because it hybridized to the translation start site of myoD [80] and contained a TCC sequence that enhanced the antisense effect by stimulating RNase H activity [81]. The antisense effect resulted in other interesting changes: the mRNA levels of myoD, myf-5 and myogenin were elevated again after 24 h, probably to reduce the backlog of regeneration. However, the mRNA levels of MRF4 and the myogenic factor inhibitory proteins Id1 and Id3 did not change. In the subsequent period, no apparent difference was observed in the myoD antisense-treated muscles except for the fact that after 28 days, more small and split fibres reflected the delayed regeneration [76]. The above results clearly show that myoD has a crucial role in muscle regeneration and indicate that this system can be a tool for investigating similar functions of the other myogenic factors [82]. The expression of connexins has also been screened in notexin-induced in vivo muscle regeneration by others. They found that only connexin 43 gap junction protein (Cx43) was present and was transiently upregulated before myoblast fusion [83]. Injection of in vitro primary muscle cells with a dye revealed more intense gap junction coupling between sparsely located pre-fusion myoblasts than between the close-fusion aligned myoblasts, implying that this tight junction protein takes part in the synchronisation of cell cycle control and the preparation for fusion to syncytial cells rather than in the fusion itself [84]. The transfection of skeletal muscle primary cultures with dominant negative Cx43 confirmed that the activity of this protein is needed for myoblast proliferation and syncytial fusion into myotubes [85]. It was later found that in regenerating muscle, the cluster of differentiated satellite cells, which represent an advanced stage of fusion in the inner part of the basal membrane, do not express Cx43, confirming that this protein does not participate actively in myoblast fusion, although it is required for the initiation of this process [86].

## 4. The Expression of Sarco/Endoplasmic Reticulum Ca^2+^ ATPases (SERCAs) during Regeneration

The SERCAs are a group of transmembrane proteins that pump Ca^2+^ from the cyto/sarcoplasm to the endo/sarcoplasmic reticulum. In striated muscle, this creates a situation in which the troponin complex allows tropomyosin to occupy the binding location of the myosin head on the actin filament and prevents actomyosin formation [87]. During excitation–contraction, the action potential spreads to the T-tubule where the dihydropyridine receptor changes conformation and opens the nearby ryanodine receptor (RyR) in the sarcoplasmic reticulum (SR) membrane. RyR then releases Ca^2+^ from the SR and the bivalent cation binds to troponin C, which changes conformation, removing tropomyosin from the myosin binding site of actin. Actin and myosin can form a cross-bridge and a new cycle begins when ATP binds to the myosin head and causes it to release the actin. The ATP is hydrolysed into ADP and phosphate by the myosin head. When phosphate is released, the myosin head binds to another position on the actin filament. Next, the ADP is released, and the myosin head pulls the actin filament toward the centre, thus shortening the sarcomere, the functional unit of contraction.

SERCA has an impact on aging and various pathological conditions [88]. There are three highly homologous SERCA genes (SERCA1–3 or ATP2A1–3 based on the human nomenclature) but only two of them, SERCA1 and SERCA2, have splice variants that are expressed in striated muscle. SERCA1a is responsible for relaxation in adult fast skeletal muscle, SERCA1b is expressed in neonatal muscle and SERCA2a is found in slow skeletal muscle and cardiomyocytes in heart ventricles [89,90,91]. SERCA1b mRNA was detected mainly in neonatal skeletal muscle and the work conducted on this system was the first to demonstrate that the protein is highly specific to myotubes and developing fibres but not to transforming or adapting muscles [92]. We also showed that SERCA1b is a major isoform in regenerating (fast or slow) muscle but is not expressed in healthy adult rat or adult human muscles where its splicing could otherwise be increased several-fold, i.e., via denervation or stretching [92,93,94,95,96,97]. Other studies [98,99] have found that the SERCA1b protein is expressed in a pathological adult human muscle condition called myotonic dystrophy type II [96]. Nevertheless, although the regenerative potential was built up in ischemic lower limb muscles in atherosclerosis obliterans, only SERCA1a–not SERCA1b–was upregulated [97].

Accordingly, neonatal 1b is the first SERCA isoform that appears shortly after myogenic regulatory factors in regenerating rat soleus and EDL muscles [28,41,100]. This predominant expression of SERCA1b indicates myotube formation and is the forerunner of the orchestrated pattern of SERCA isoforms found characteristically at both messenger and protein levels during muscle regeneration. This motif is strongly dependent on the progress of innervation and the type of innervation (fast or slow) and is expressed in developing myofibres before they become fast or slow. This means that the start of innervation will switch the splicing of SERCA1 transcript from neonatal to adult fast-type mRNA during both fast and slow muscle regeneration [28,100]. The switch between SERCA1a and SERCA2a occurs during slow fibre regeneration, when innervation is more established [77,101] and the fast SERCA1a reduces while the slow SERCA2a increases, becoming exclusive (or at least predominant) in slow muscle [28,41]. Interestingly, the fast twitch muscle follows an alternate motif that starts with a moderate elevation followed by a decline in SERCA2a expression, paralleled with that of SERCA1b, before an increase and predominance of SERCA1a as innervation progresses [100].

The local and regional effects of notexin-induced necrosis were reviewed briefly in the second paragraph of the introduction. However, there are limited observations on the same aspects during the regeneration of the soleus. Inflammatory cells were abundant in haematoxylin–eosin-stained cross-sections of the lower hind limb but regeneration of other muscles was not observed. The relative intactness of other muscles maintained the passive movement of the soleus and this was beneficial for regeneration [40]. In the longer term, the soleus reached the size of the control fibre by day 21–28 and regenerated into the same uniformly slow phenotype as the untreated age-matched control after six months [41].

The change in SERCA levels appears to be similar to the innervation-dependent expression of myosin isoforms during regeneration, especially in the predominantly slow soleus muscle [33,102]. This prompted us to investigate whether the level of the slow muscle type, SERCA2a, is co-regulated with that of the heavy chain of the corresponding slow myosin isoform, MyHC1. In bupivacaine-induced regeneration of rat soleus, MyHC1 expression is upregulated by the innervation of the slow type. The upregulation can be prevented by transfecting dominant negative Ras N17 (dnRas) into the regenerating muscle fibres. Interestingly, when bupivacaine regeneration was induced in denervated rat soleus, the transfection of Ras V12 S35, which influences the downstream MAPK pathway, restored MyHC1 expression in the fibres while the other Ras mutants did not [29]. This suggests that a distinct well-defined pathway mediates the nerve effect on MyHC1 expression during regeneration. It was of interest to determine whether the same pathway could also function in the upregulation of SERCA2a. However, the expression of SERCA2a was not dependent on slow-type innervation like MyHC1, and neither was it influenced by the transfection of Ras mutants regulating MyHC1 expression [40]. Therefore, the slow types of SERCA2a and MyHC1 are not co-regulated by innervation and Ras. A similar conclusion was drawn after experiments using calcineurin inhibitor Cain/cabin [103]. The calcineurin–NFAT pathway was also found to mediate the effect of slow-type innervation on the expression of MyHC1 in bupivacaine-induced regeneration of rat soleus [104,105]. However, while transfection with Cain inhibited MyHC1, it did not prevent the expression of SERCA2a during the notexin-induced regeneration of rat soleus [103]. This again confirmed that SERCA2a expression is regulated by pathway(s) separate from those that control slow myosin expression. It was later reported that slow myosin does not depend on slow-type innervation or calcineurin in muscles other than the soleus [106]. However, we did not find a similar report for SERCA2a. Thus, the question of what regulates SERCA2a expression remains. First, SERCA2a regulation is not exerted as much at the transcript level as MxHC1 expression [29] but clearly has more components [107]. One of these complex effects is the passive stretch that works in selective denervation of the soleus but not in ischiadic (hind limb) denervation. To illustrate this, SERCA2a was upregulated in the selective denervated vs. ischiadic denervated regenerating soleus [40]. Similarly, SERCA2a was upregulated in dnRas-transfected fibres of innervated regenerating muscle, which were obviously stretched by the overwhelming innervated fibres [40].

## 5. Novel Muscle Growth Regulation Revealed Using Molecular Acupuncture during Regeneration Takes after a Scale-Free Network

Transfection of regenerating soleus with dnRas resulted in a phenomenon that, to the best of my knowledge, has not been reported previously [108]; besides inhibiting the expression of slow MyHCI in the transfected fibre [29], dnRas resulted in a non-fiber-autonomous effect. Although only 20–30 fibres out of the total (approximately 2500) were transfected and expressed the transgene in a few of their myonuclei [109], the fibre size (cross-sectional area, CSA) in the entire muscle increased by 20–30% between days 7–12 of regeneration compared with the control regenerating soleus transfected with empty vector [108]. The sizes of transfected fibres were not different from those of non-transfected ones in the same muscle and showed no morphological or histochemical differences. However, after 21 days, when the full fiber size had nearly been reached, the increase in growth was attenuated; therefore, it did not exceed that of the control. The augmentation of fibre growth started 4 days after transfection and finished by day 21 of regeneration, and was apparently mediated by the autocrine–paracrine effect. One of the factors that might orchestrate the effect of transfection was interleukin 4 (IL4) since the perimuscular injection of its antibody stopped the augmentation of CSA increase. However, the IL4 antibody did not decrease the fibre size below the level of the non-transfected regenerating soleus, suggesting that the cytokine has a role in the augmentation of fibre growth only and not in the normal regeneration process. Interestingly, higher IL4 levels were detectable by immunohistochemistry only around the transfected fibres and not by immunoblot of the whole muscle, supporting local importance instead of a widespread systematic one. IL4 is secreted by growing myotubes and acts on myoblast receptors, stimulating their accretion [110,111]. As IL4 expression is regulated by the calcineurin–NFAT pathway [110,112], the calcineurin inhibitor peptide Cain/cabin was co-transfected with dnRas in the regenerating soleus [108]. This prevented the augmentation of CSA increase, suggesting that the calcineurin–NFAT–IL4 pathway was activated by Ras inhibition. This revealed a scenario in which the calcineurin–NFAT and Ras pathways act in unison to control the fibre growth of regenerating soleus muscle. Based on this, Ras inhibited calcineurin but the transfected dnRas increased calcineurin activity and growth augmentation.

Although Ras and calcineurin are members of two important pathways, to the best of my knowledge, there is little information on their connection in skeletal muscle. In neonatal cardiac myocytes, they appeared to be synergistic and not antagonistic since the constitutively active Ras increased the activity of NFAT3 [113]. In these cells, Ras acted on NFAT3 activity upstream of calcineurin and similarly, dominant negative Ras inhibited the stimulated NFAT nuclear location and transcriptional activity [113]. Essentially similar coordinated functions have been found in vivo between calcineurin–NFAT and the Ras-stimulated MAPK–ERK pathway in cardiac hypertrophy in transgenic mice [114]. Since this interaction has not been determined in muscle cells, we inhibited the main pump that regulates sarcoplasmic Ca^2+^ in regenerating muscle fibres. SERCA1b is a major isoform in neonatal and regenerating muscles [92,96,98]. Transfection of SERCA1b-shRNA-expressing vector into the fibres of regenerating soleus resulted in the augmentation of CSA increase in both transfected and non-transfected fibres of the whole muscle [115] in a manner similar to that of dnRAS [108]. Growth stimulation was also dependent on calcineurin and IL4, as it was abolished by the co-transfection of Cain and the perimuscular injection of IL4 antibody [115]. This indicates the pivotal role of the calcineurin–NFAT–IL4 pathway in fiber growth augmented by both dnRas and SERCA1b silencing in regenerating soleus muscle.

Although IL4 appeared to be essential in molecular acupuncture-like stimulation of regeneration, this cytokine was only present in some transfected fibres. This suggests that IL4 is not a long-range diffusing factor that mediates growth stimulation throughout the entire regenerating muscle. Nevertheless, autocrine–paracrine mechanisms may also include cell interactions. Non-myogenic fibro-adipogenic progenitor (FAP) cells were recently found to have a pivotal role in orchestrating muscle regeneration, as reviewed in [15,16]. In addition to their numerous pro-myogenic effects, they also clear out debris, reproduce scaffold and, interestingly, escape from dormancy in response to IL4. This cytokine also inhibits adipogenic differentiation of FAPs [116], which is known to give way to pro-myogenic events. Therefore, it is feasible to suggest that dnRas or SERCA1b silencing via the calcineurin–NFAT–IL4 pathway might stimulate muscle regeneration by activating FAPs. The entire increase in regeneration is obviously helped by other cells, growth factors and developmental pathways, but most of these can also be associated with the central role of FAPs [15,16].

It is worth emphasizing that silencing SERCA1b in a small fraction of fibres had dramatically different effects compared with knocking out the SERCA1 gene in mice, as the latter resulted in perinatal lethality [117]. However, it showed a somewhat similar phenomenon to the muscle-specific knockout of SERCA-inhibiting micropeptide myoregulin, which resulted in enhanced exercise performance in mice [118].

**Figure 2:**
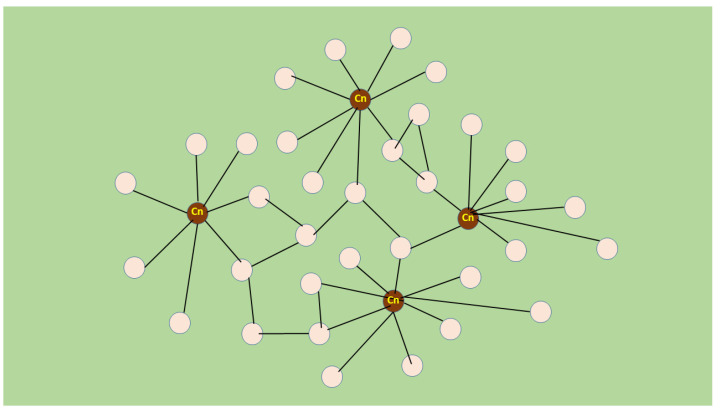
Scale-free network in molecular-acupuncture-stimulated muscle regeneration. Myonuclear domains (Md) are depicted as nodes (illustrated with circles) and lines indicate links between the nodes. Md transforms into a “hub” (a high-grade node with many links) when transfected with dnRas or SERCA1b shRNA, probably because of its increased calcineurin (Cn) activity. The links between hubs and non-transfected Mds might be formed by autocrine–paracrine factors like IL4 and (non-myogenic) cells like FAPs. Cn and brown colour indicate Mds with hub-creating calcineurin activity. This representation is not intended to show the entire operation of the growth network; it is only meant to reflect the driving process.

Remarkably, only a few (1–4) myonuclei were transfected per fibre in the muscle belly [109]. The total number of myonuclei in a regenerating fibre was estimated to be several-fold higher than those transfected [109,119]. Therefore, the growth stimulation must have been initiated from a small minority of myonuclei [108,109,115]. However, the myonuclei surrounded by zones form a myonuclear domain, which is a functional unit of gene expression within the multinucleated fibre [120]. The plasmids have to reach the nuclei and transfect the myonuclear domain in order for expression to occur. The rare transfections of nuclei, such as that which occurs in regenerating rat soleus, resemble the effect of acupuncture [121]. However, this time the manipulation was caused by molecular inhibition such as by the dominant negative Ras or by SERCA1b silencing instead of a special needle. Thus, this phenomenon can be described illustratively as molecular acupuncture of regenerating skeletal muscle. Furthermore, if one wants to depict the relationship between transfected and non-transfected myonuclear domains in the above-described stimulated fibre growth, it is appealing to adapt a scale-free network, at least for the initial events (Figure 2) [122,123]. In this network, the nodes are not identical since some have a higher number of links than others. These high-degree nodes become hubs, unlike the other low-degree nodes that are abundant. The hub quality is affected by transfected myonuclei because of their calcineurin activity, while the non-transfected nuclei remain as low-degree nodes. The regenerating muscle is probably a self-organizing network and creates new hubs from low-degree nodes using other features as none of the transfected fibres show hypertrophy compared with the non-transfected fibres. Independent regulation of myonuclei for gene expression has been shown along multinucleated fibres and is suggested to be organized in a stochastic pattern [124]. However, to the best of my knowledge, no network-like interaction between myonuclear domains has been reported in different myofibers. This suggests that the regeneration system discussed here may fill a gap in the study of biological networks.

## 6. Conclusions

A synchronized in vivo muscle regeneration study revealed a novel network of growth regulation. The network was stimulated by changing the equilibrium between two major signal transduction systems controlled by Ras and calcineurin within the same muscle fibre. Shifting this dynamic balance by stimulating calcineurin influences other fibres, probably in an autocrine–paracrine way, and augments fibre growth in the entire regenerating muscle. The reason why such an effect has not been reported before might be because it was not possible to detect growth stimulation reliably in unsynchronized and partial regenerations where the fibres did not grow evenly; therefore, comparison with the control was hampered. Thus, notexin-induced soleus regeneration in rats offers a unique in vivo system for studying novel effects of signal pathway interactions and apparently represents an example of scale-free networks of signalling in skeletal muscle. It would be interesting to determine whether co-transfection with dnRas can interfere with SERCA1b shRNA-induced fibre growth amelioration and whether transfection with the calcineurin inhibitor Cain alone can have any effect. Experiments in this area are ongoing.
